# Transcriptome analysis reveals *SALL4* as a prognostic key gene in gastric adenocarcinoma

**DOI:** 10.1186/s43046-022-00108-5

**Published:** 2022-03-14

**Authors:** Ranjan Jyoti Sarma, Sarathbabu Subbarayan, John Zohmingthanga, Saia Chenkual, Thomas Zomuana, Sailo Tlau Lalruatfela, Jeremy L. Pautu, Arindam Maitra, Nachimuthu Senthil Kumar

**Affiliations:** 1 Department of Biotechnology, Mizoram University, Aizawl, Mizoram 796 004 India; 2 Director, Zoram Medical College, Falkawn, Mizoram 796005 India; 3 Department of Surgery, Civil Hospital Aizawl, Aizawl, Mizoram 796 001 India; 4 Department of Medical Oncology, Mizoram State Cancer Institute, Aizawl, Mizoram 796017 India; 5 National Institute of Biomedical Genomics, Kalyani, West Bengal 741251 India

**Keywords:** Stomach adenocarcinoma, RNA-Seq, Differentially expressed genes, Poor survival, Biomarker

## Abstract

**Background:**

Stomach adenocarcinoma (STAD) dominates 80–90% of gastric cancer (GC). Over the years, it has been realized that the identification of the genes responsible for gastric carcinogenesis is essential to understand the biomarker discovery.

**Methods:**

This study aims to identify candidate genes for biomarker discovery in STAD. RNA-Seq was performed on three paired tumor-normal and one unpaired tumor samples from four GC patients and investigated for differentially expressed genes (DEGs) using DESeq2. Gene set enrichment analysis were performed. The DEGs were compared with two STAD microarray datasets available on Gene Expression Omnibus (GEO) database. Survival study (OS) were performed using KM-Plotter on the common genes between all the datasets.

**Results:**

Totally, 148 DEGs were identified, wherein 55 genes were upregulated and 93 genes were downregulated with |log2foldchange| > 1 and Benjamini-Hochberg (BH) Adjusted *P* value < 0.01. Cell adhesion molecule (CAM) Pathway was found to be the most significant among the upregulated genes. Gastric acid secretion and mineral absorption pathways were the most significant pathways among the downregulated genes. Comparison with two GEO datasets followed by OS analysis revealed two upregulating genes, *APOC1* and *SALL4* with prognostic significance.

**Conclusion:**

Upregulation of *APOC1* is associated with marginal overall survival (OS) and *SALL4* over-expression was associated with the poor OS using KM-Plotter during 5 years data period. Our study suggests that *SALL4* could be a promising biomarker candidate in STAD.

**Supplementary Information:**

The online version contains supplementary material available at 10.1186/s43046-022-00108-5.

## Background

Gastric cancer symptoms are misunderstood often with the other stomach complications, which leads to diagnosis at an advanced stage and poor treatment due to cancer heterogeneity [1]. STAD dominates the major type of GC; the second deadliest cancer type worldwide and associated with a poor survival rate [2]. Eighty to 90% of GC cases belong to STAD are primarily associated with intestinal metaplasia; however, surgical resection is still a promising curative treatment [3, 4]. However, the identification of biomarkers to predict the outcome of the particular treatment is another challenging task and equally crucial.

Biomarker discovery enables the understanding of the disease diagnosis, prognostic events, and selection of the treatment strategies. CEA, CA19-9, and CA72-4 belong to carcinoembryonic antigens and are currently used biomarkers in clinical setup despite having low specificity for evaluating diagnosis and the prognosis of GC [5, 6].

Recent advances in transcriptome array and whole transcriptome sequencing have generated a tremendous amount of data and are being deposited in the publicly available databases. Experimental data could be compared with such publicly available data to increases the authenticity of the study [7]. Comparison of tumor gene expression profiles with the normal tissues is crucial for investigating differentially expressed genes (DEGs), different pathways which enable researchers to understand mechanisms of cancer development, progression, and response to the targeted therapies [8].

Identification of such biomarkers is crucially important to monitor the patient health after treatment and the after-effects during the recovery period could be avoided. This study aims to identify the potential pathways in GC and the genes involved in STAD using transcriptomics approach.

## Methods

### Sample collection and total RNA isolation

Paired tumor-normal samples from four GC patients were obtained after surgery and the samples were immediately transferred into RNAlater (Thermo Fischer Scientific, USA) solution and stored at – 80 °C temperature. The participants did not have previous GC history. High quality total RNA was extracted from adjacent normal and Tumor Tissue samples using the PureLink RNA mini kit (Ambion, Inc.) as per the manufacture’s protocol. Agilent RNA 6000 Nanochips in 2100 Bioanalyzer (Agilent, Inc) was used to check the quality of isolated total RNA and quantitation was done by Qubit using the Quant-iT RNA assay kit broad range and NanoDrop spectrophotometer (Thermo Ficher Scientific, USA). A summary of the clinical data of the patients is given in the Supplementary Table 1.

### Library preparation, sequencing, and data processing

The sequencing library was prepared using Illumina TruSeq Stranded Total RNA Library preparation kit (Illumina Inc., USA) from the samples bearing RNA Integrity Number (RIN) ≥ 6. Three paired tumor-normal and one unpaired tumor sample from four GC patients possessed RIN ≥ 6 (Supplementary Table 2) were further processed for RNA-Seq. Briefly, 1 μg of total RNA was taken for library preparation and rRNAs were removed before fragmentation and adapter ligation. cDNA library containing first and second-strand cDNA was synthesized from rRNA-depleted fragmented total RNA, both ends of cDNA were repaired and adapters were ligated, and final libraries were enriched using limited cycle PCR. The yield of cDNA libraries was quantified using Qubit dsDNA HS assay kit (Invitrogen, USA), size distribution and quality of the cDNA libraries were assessed using a High Sensitivity chip in Bioanalyzer (Agilent Technologies, USA), respectively. Quantitative Real-Time PCR was used to quantify the final library. The clusters were generated on a cBot cluster generation system (Illumina) and paired-end 2 × 100 bp sequencing was performed in Illumina HiSeq-2500 (Illumina Inc., USA).

### Data processing and analysis

The raw data were checked with FASTQC tool [9]. The low-quality bases and adapters were removed using Trimmomatic v0.38 [10]. The average number of reads after trimming was reduced to 16 million reads from 18 million reads (Supplementary Table 3). The raw reads were aligned to the human reference genome (GRCh38) using STAR aligner [11]. The generated BAM files were sorted by coordinates using Samtools [12]. The transcript counts were estimated using featureCount tool [13] with GRCh38 version-based gene annotations. The low expressed genes which did not have more than 15 counts per million (CPM) reads in all the samples were removed from subsequent analysis. The null hypothesis for the experiment was that there was no difference in expression between the paired normal samples (log2foldchange is equal to Zero). DESeq2 [14], a R/Bioconductor [15] package which uses the Wald test for hypothesis testing was used to produce the gene list after ranked by *P* value and Adjusted *P* value for multiple testing using the Benjamini-Hochberg method. The log-transformed normalized counts were used to calculate the principal component analysis (PCA) to assess normalized expression pattern before differential expression test between tumor and adjacent normal tissues. |Log2foldchange| > 1 (rejection of null hypothesis) and adjusted *P* value < 0.01 were set as the criteria to get the significantly upregulated and downregulated genes. “ggplot2” packages were used to generate the volcano plot.

### Functional enrichment analysis

EnrichR (https://maayanlab.cloud/Enrichr/) was used to study the Gene Ontology (GO) for the Biological process (BP), Molecular function (MF) and Cellular component (CP). Similarly, KEGG Pathway and Disease-Gene Association (DisGeNET) analysis using EnrichR. EnrichR uses Fisher-exact test to calculate the *P* value and adjusted *P* value using Benjamini-Hochberg method for correction for multiple hypotheses testing [16–18]. The Adjusted *P* value < 0.05 was considered statistically significant for both the GO analysis and the pathway enrichment analysis to identify the significant GO terms and pathways.

### GEO2R analysis and comparison with RNA-Seq data

For comparative study with gene expression data from other STAD patients, microarray datasets GSE19826 and GSE79973 from Gene Expression Omnibus (GEO) were used (Table 1). The differential gene expression was analyzed using GEO2R (https://www.ncbi.nlm.nih.gov/geo/geo2r/). We have identified the DEGs from the two datasets using the criteria of |log2folchange| > 1 to get upregulated and downregulated genes and adjusted *P* value < 0.01 as statistically significant. The Venn Diagram tool (http://bioinformatics.psb.ugent.be/webtools/Venn/) was used to find out the common genes from the DEGs identified from the RNA-Seq experiment and two GEO Datasets.

**Table 1:** GEO Datasets selected for comparison with the RNA sequencing data from GC patients from Mizoram

Dataset ID	Tumor	Adjacent Normal	Total Sample	Platform	Citation
GSE19826	12	15	27	GPL570	[19]
GSE79973	10	10	20	GPL570	[20]

### Overall Survival (OS) analysis

The “Kaplan-Meier plotter” (https://kmplot.com) tool was used to analyze the overall survival of the key overlapping genes. KM-Plotter utilizes Cox proportional hazards regression analysis and calculates the log-rank *P* value [21]. KM-Plotter uses background databases derived from manually curated clinical data as well as the gene expression data from GEO datasets. The analysis was restricted to 60 months OS at cancer stage III. Log-rank *P* value < 0.05 was set to be statistically significant.

## Result

### Identification of the DEGs from RNA-Seq data

**Fig. 1:**
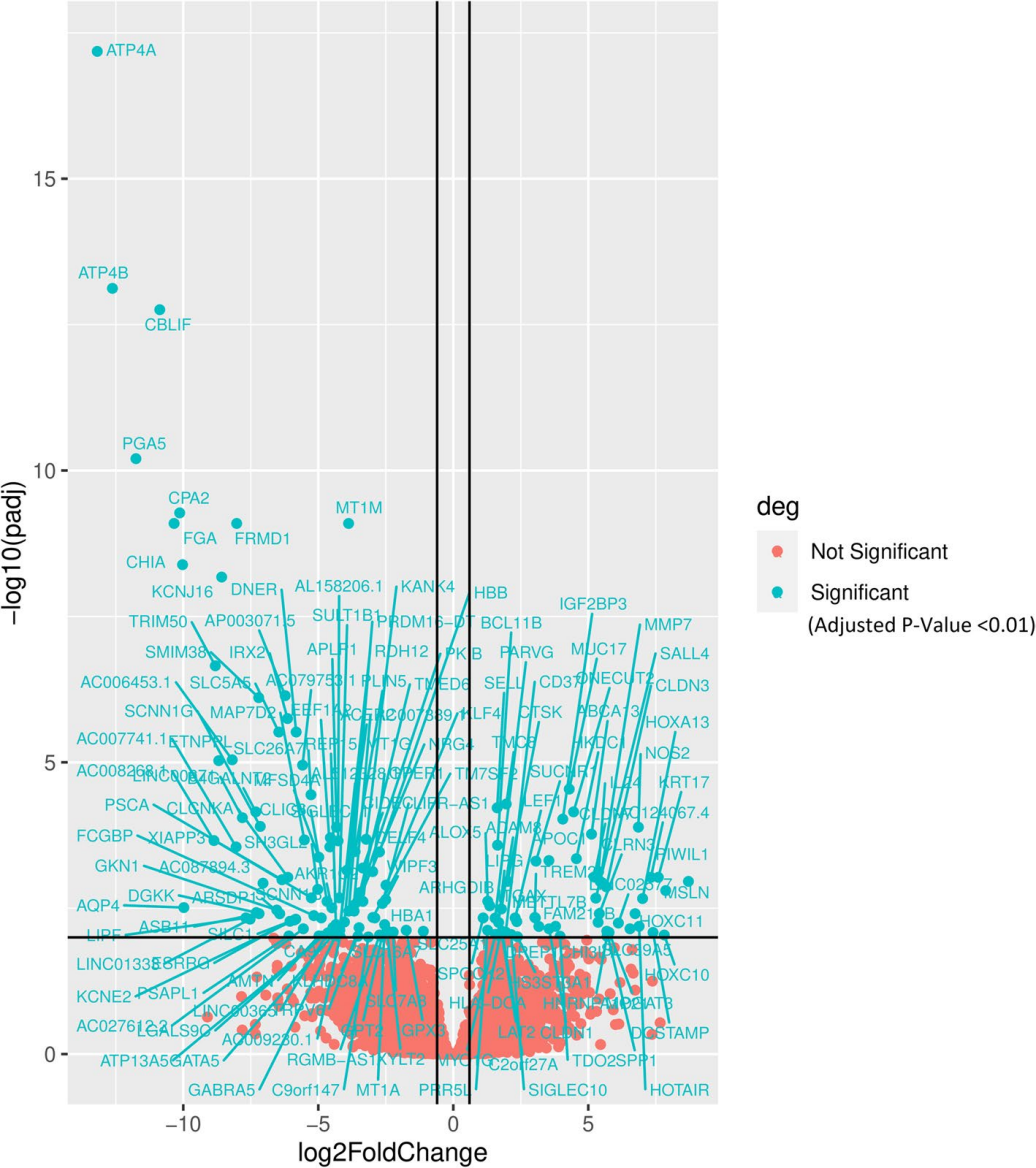
Volcano plot showing the significant different ially expressed genes (DEGs). The adjusted *P* value less than 0.01 was set as statistically significant. log2foldchange > 1 is set to be upregulated and log2folchange < − 1 is set to be downregulated

Principal component analysis (PCA) was performed to understand the variation in the normalized expression pattern between the tumor and the adjacent normal samples. PC1 and PC2 were observed to be 52% and 31% variance, respectively (Supplementary Figure 1). Total 148 genes showed highly significant differential expression with adjusted *P* value < 0.01 wherein 55 genes were upregulated (log2foldchange > 1) and 93 genes were downregulated (log2foldchange < − 1) (Fig. 1). The upregulated and downregulated genes with adjusted *P* value and log2foldchange are provided in Supplementary Table 4A and B.

### Functional enrichment of the DEGs

The Gene Set Enrichment Analysis for upregulated and downregulated genes was performed for Gene Ontology terms for biological process, molecular function, and cellular component (Table 2). Pathway analysis of the upregulated gene set identified the Cell Adhesion molecule (CAM) pathway as the most significant pathway with adjusted *P* value of 0.004517. Among the downregulated genes, the Gastric acid secretion pathway and Mineral absorption pathway were found to be the most significant pathways with adjusted *P* values 0.001302 and 0.002315 (Table 3).

**Table 2:** Genes enriched in the Gene Ontology (GO) terms

Relative Gene Expression	BP	MF	CP
Upregulated	Positive regulation of bone resorption (GO:0045780) *0.001907*	--	--
	Cellular component maintenance (GO:0043954) *0.001907*		
Downregulated	Sodium ion homeostasis (GO:0055078) *0.003497*	P-type potassium transmembrane transporter activity (GO:0008556) *0.034526*	--
	Bicarbonate transport (GO:0015701) *0.006706*	Ligand-gated cation channel activity (GO:0099094) *0.034526*	
	Inorganic cation import across plasma membrane (GO:0098659) *0.006706*	P-type proton-exporting transporter activity (GO:0008553) *0.034526*	
	Potassium ion import across plasma membrane (GO:1990573) *0.007014*	Ligand-gated sodium channel activity (GO:0015280) *0.034526*	
	Metal ion homeostasis (GO:0055065) *0.007014*	Channel activity (GO:0015267) *0.037584*	

The Adjusted *P*-Value are mentioned in italics below the GO terms

*BP* Biological Process, *MF* Molecular Function, *CP* Cellular Component

--: No GO terms enriched with Adjusted *P*-Value <0.05

**Table 3:** Genes enriched in the KEGG pathway

Relative Gene Expression	Pathways	Genes	Adjusted ***P***-Value
Upregulated	Cell Adhesion Molecule	*CLDN7, SELL, CLDN4, HLA-DOA, CLDN1*	0.004517
Downregulated	Gastric Acid Secretion	*ATP4A, ATP4B, KCNE2, KCNJ16, SLC26A7*	0.001302
	Mineral absorption pathway	*MT1A, TRPV6, MT1M, MT1G*	0.002315

In the Disease-Gene Association analysis**,** the upregulated genes were found to be associated with stomach neoplasm (adjusted *P* value: 0.0001783), malignant neoplasm of stomach (adjusted *P* value: 0.002834), and tumor progression (adjusted *P* value: 0.004874). On the other hand, hypokelmia, characterized by less K^+^ absorptions are found to be significant (adjusted *P* value: 0.04754) among the downregulated genes (Table 4).

**Table 4:** Genes enriched in DisGeNET disease terms

Relative Gene Expression	Disease Terms	Associated Genes	Adjusted ***P***-Value
Upregulated	Stomach Neoplasm	*HOTAIR, PIWIL1, MMP7, NOS2, MSLN, CLDN1, CLDN3, SELL, ALOX5, CLDN7, ARHGDIB, SPP1, IGF2BP3*	0.001783
	Malignant neoplasm of Stomach	*HOTAIR, MMP7, MUC17, LEF1, IL24, MSLN, CLDN1, CLDN3, KRT17, SELL, ALOX5, CLDN7, APOC1, SPP1, DPEP1, CHI3L1, ADAM8, IGF2BP3, HLA-DOA*	0.002834
	Tumor Progression	*HOTAIR, PIWIL1, MMP7, BCL11B, NOS2, MUC17, IL24, MSLN, CLDN1, CLDN3, CTSK, ALOX5, SALL4, CLDN7, ARHGDIB, SPP1, CHI3L1*	0.004874
Downregulated	Hypokalemia	*SCNN1G, SCNN1B, CLCNKA, SLC26A7*	0.04758

### Identification of DEGs from GEO datasets and comparation with RNA-Seq data

**Fig. 2:**
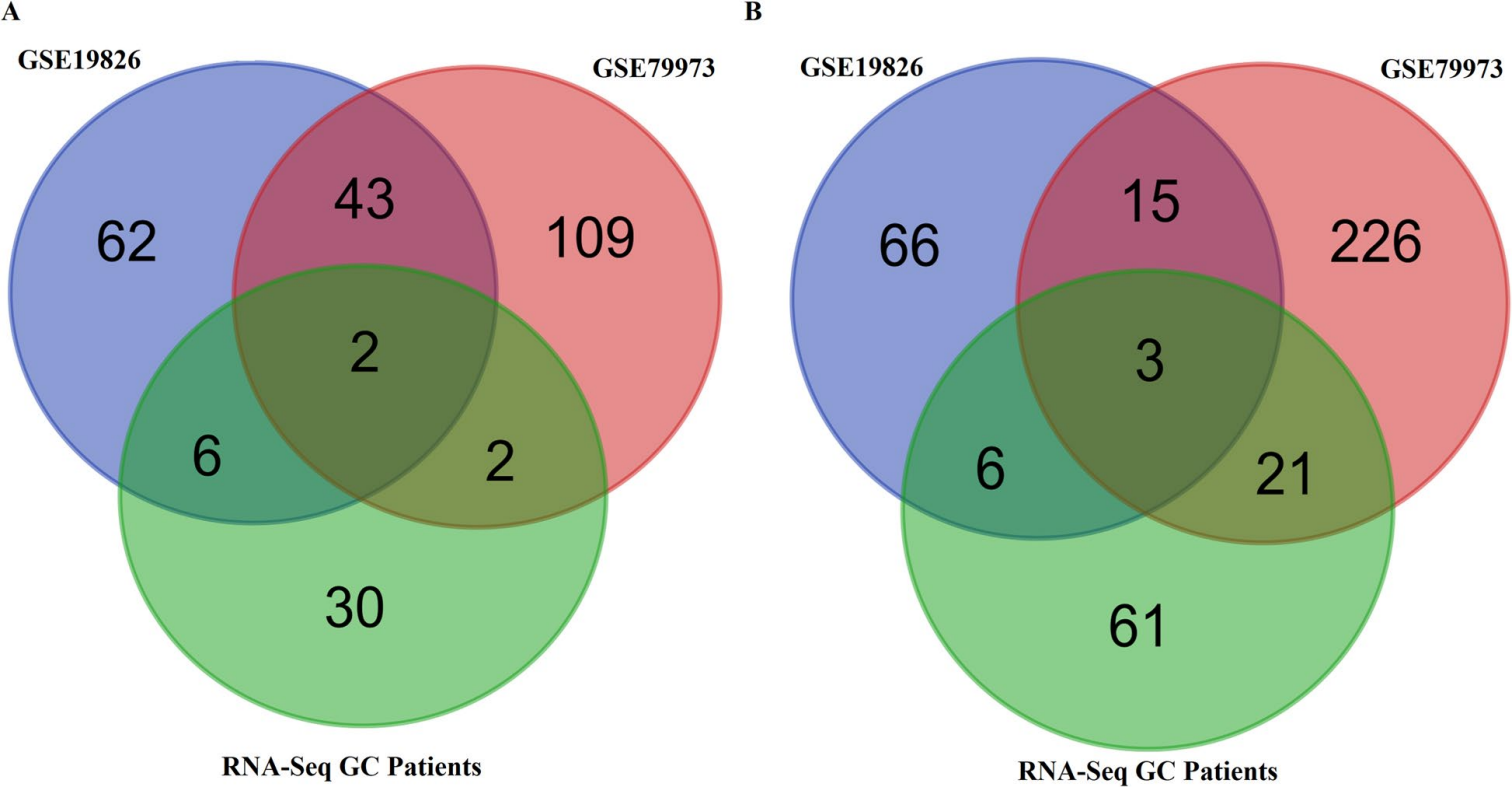
Comparison of the three datasets. Common genes among **A** upregulated genes and **B** downregulated genes. The figures are generated in http://bioinformatics.psb.ugent.be/webtools/Venn/

In GSE19826, we found 145 upregulated and 103 downregulated genes. Similarly, in GSE79973, we found 210 genes as upregulated and 343 genes as downregulated. The DEGs obtained from two datasets from the GEO database using GEO2R as well as the DEGs from our study were used to find out the most common genes among the upregulated and downregulated genes. *APOC1*, *SALL4* were the commonly upregulated genes and *PSAPL1*, *CLIC6*, *TRIM50* were the commonly downregulated genes in all the three datasets. Comparison of the datasets for upregulated and downregulated genes is represented as Venn diagram (Fig. 2A, B). The genes common among the three datasets are also provided in detail in the Supplementary Table 5A-B.

### Survival probability

**Fig. 3:**
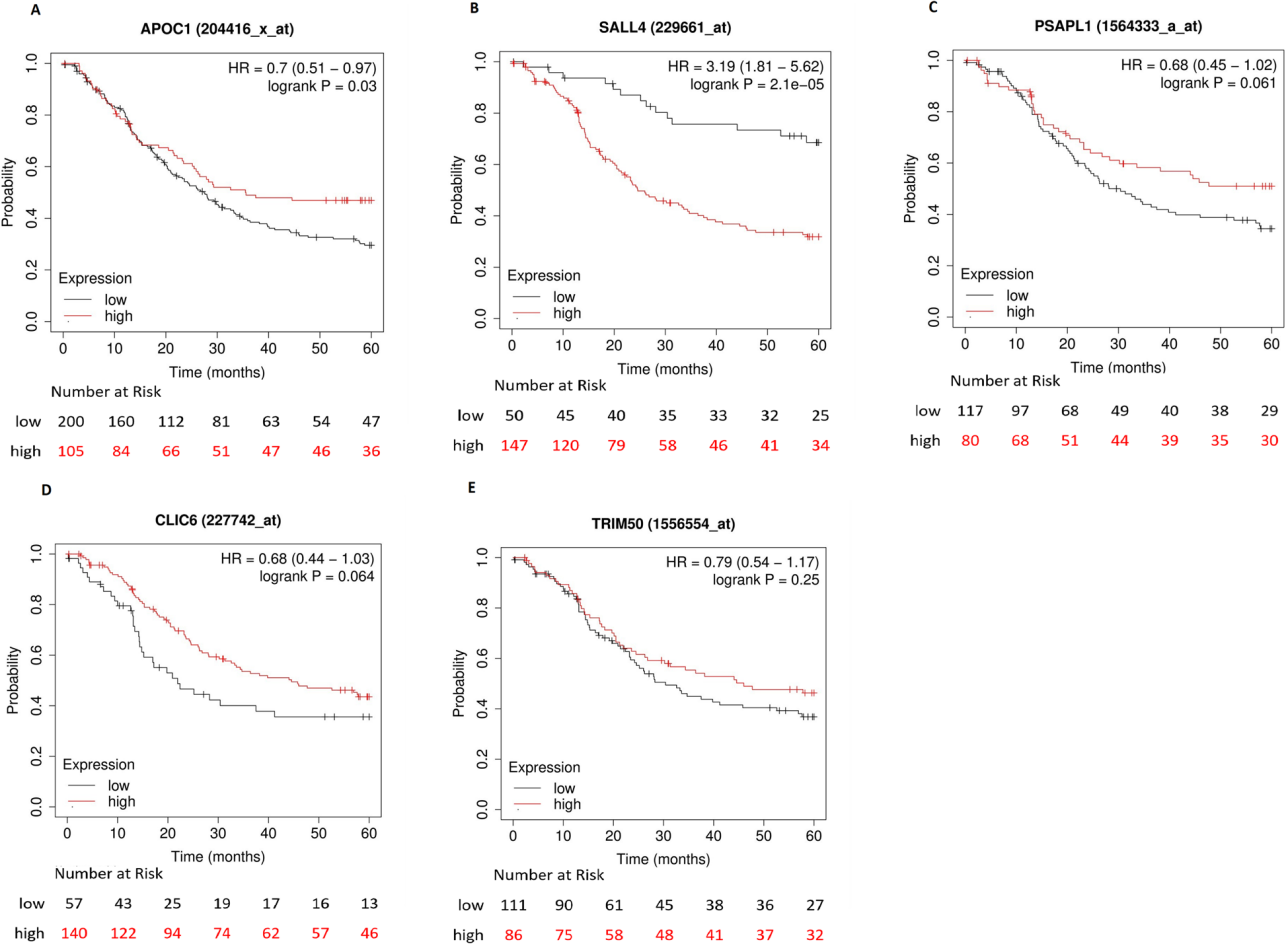
KM-Plot for the survival analysis using the common genes. **A** *APOC1.* **B** *SALL4.* **C** *PSAPL1.* **D** *CLIC6.* **E** *TRIM50.* The plots are generated on the basis of the available data on KM-plotter database of 5-year survival data of gastric cancer at stage III. The red plot represents the high expression and black plot represents low expression of the genes in the cohort

The KM plotter was used to investigate the genes for overall survival and the final analysis was run based on 305 patients’ data for the *APOC1* gene and 197 patients’ data for *SALL4*, *PSAPL1*, *CLIC6*, and *TRIM50* genes. It was found that high expression of *APOC1* is associated with marginal better OS with log-rank *P* value 0.03 and hazard ratio 0.70. *SALL4* upregulation is significantly associated with very poor OS with log-rank *P* value 0.000021 and hazard ratio 3.19 (Fig. 3A, B). The median OS associated with *APOC1* was found 35.5 months in the high expression cohort and 27.4 months in the low expression cohort. The median OS associated with *SALL4* was 44.07 months in the low expression cohort and 13.04 month in the high expression cohort. The OS associated with *PSAPL1*, *CLIC6*, and *TRIM50* genes were found to be insignificant with log-rank *P* values of 0.061, 0.064, and 0.25, respectively (Fig. 3C–E).

## Discussion

In gastric adenocarcinoma, we have analyzed patients’ tumor samples along with adjacent normal collected after radical resection and 148 highly significant (adjusted *P* value < 0.01) differentially expressed genes were identified using RNA-Seq data. We have analyzed for functional enrichment of the upregulated and the downregulated genes to understand the underlying pathways and GO for the underlying biological functions. The resulting gene set from the RNA-Seq experiment was compared with the gene expression data of two microarray datasets: GSE19826 (12 tumor and 15 adjacent normals) and GSE79973 (10 tumor and 10 adjacent normals).

Pathway analysis of upregulated genes has identified CAM pathway as significant (adjusted *P* value: 0.004517). *CLDN7*, *SELL*, *CLDN4*, *HLA-DOA*, and *CLDN1* genes were enriched in the CAM pathway. *CLDN1*, *CLDN4*, and *CLDN7* belong to the Claudin family and involve in tight junction and are often upregulated in neoplastic tissue. Among the family of claudins, *CLDN4* and *CLDN7* are more often to be upregulated in stomach cancer as well as other malignancies [22]. Moreover, several other reports have confirmed their upregulation in cancer of various sites [23]. Among the downregulated genes, the most significantly enriched pathways were the gastric acid secretion (adjusted *P* value: 0.001302) and mineral absorption pathways (Adjusted *P* value: 0.002315)*. ATP4A*, *ATP4B*, *KCNE2*, *KCNJ16*, and *SLC26A7* genes were enriched in the gastric acid secretion pathway. These genes are commonly downregulated genes in gastric adenocarcinoma as reported by many previous studies [24, 25]. *ATP4A* and *ATP4B* downregulation is often associated with DNA methylation and *ATP4B* could also be a potential biomarker of gastric adenocarcinoma [26]. *MT1A*, *TRPV6*, *MT1M*, and *MT1G* genes were enriched in the mineral absorption pathway.

It is always essential to know or predict the association of disease with the gene or a gene set. DisGeNET is such a database that has a collection of genes as well as their associated disease derived manually from popular databases like Comparative Toxicogenomic Database (Human Subset), UniProt, ClinVar, ClinGen OMIM, and Orphanet as well as extensive text mining data [27]. EnrichR offers the facility to analyze the gene sets against DisGeNET on the web to quickly understand the associated disease with such genes. Most interestingly, the upregulated genes enriched in two stomach related diseases: stomach neoplasm with adjusted *P* value of 0.001783 and malignant neoplasm of the stomach with adjusted *P* value of 0.002834. Moreover, the few upregulated genes were also enriched in the tumor progression with adjusted *P* value of 0.004874. *SPP1*, *MSLN*, *HOTAIR*, *CLDN1*, *CLDN3*, *CLDN7*, *ALOX5*, and *MMP7* genes were found to be common in all these three disease terms. The significant disease term enriched in the downregulated genes was hypokalemia with adjusted *P* value of 0.04758. However, hypokalemia, characterized by low K^+^ level, association with GC is not reported much worldwide. Extensive studies might be required to find out the relation of Hypokalemia with GC development. The pathways and the disease terms along with the genes involved are provided in the Table 2.

Comparison of the gene set resulted from the RNA-Seq data with the GEO datasets has allowed us to find out the most common genes. This comparison carries the overall authenticity of differentially expressed genes in gastric adenocarcinoma. We found the genes *APOC1* and *SALL4* common in all the datasets used for the comparative study which has an impact on OS in GC patients. The expression levels of the common genes are provided in Supplementary Figure 2. Upregulation of both the *APOC1* and *SALL41* has an opposite effect on the OS. Upregulation of *APOC1* is associated with marginal better OS, while upregulation of *SALL4* has extremely poor OS. The relative expression of *APOC1* which codes for apolipoprotein C1 was detected upregulated in the serum of a large number of GC patients and was identified as a potential biomarker candidate [28]. *SALL4* encodes for zinc finger transcription factor is reported to have a role in GC development and found to be overexpressed in GC cases [29]. It was elucidated that upregulation of *SALL4* involves GC by activating the Wnt/β-catenin signaling pathway. *SALL4* was also found to be associated with poor survival in TCGA (The Cancer Genome Atlas) STAD data [30].

In summary, although we found significantly large numbers of DEGs, the bioinformatics analysis has enabled us to find out the promising factors (genes) that have a probable impact on gastric carcinogenesis. We have identified important pathways that were altered in GC. The Disease-Gene association study provides the authenticity of the upregulated gen es with the associated disease. At last, comparative study of RNA-Seq with the other STAD patient microarray data has enabled us to find out the most common genes among them and the survival study has converged our focus on the *SALL4* gene. However, in-depth study with patient’s clinical data in larger cohorts and a higher sequencing depth is required to explore more hidden biological information in STAD that may enable the researcher to discover new biomarkers.

## Conclusion

In conclusion, we found the genes associated with CAM pathway, gastric acid secretion, and mineral absorption pathways altered their expression in STAD samples. *APOC1* and *SALL4* genes were upregulated in STAD tissue and might affect the disease prognosis. The KM-Plotter analysis revealed that the upregulation of *SALL4* is associated with overall poor survival in GC patients and may act as a promising prognostic biomarker.

## Supplementary Information

**Additional file 1: Supplementary Table 1.** Summary of Clinical information of patients included in the study. **Supplementary Table 2.** Concentration, Purity, and Integrity of Total RNA extracted from Adjacent Normal (D) and Tumor (T) tissues. **Supplementary Table 3.** Summary of RNA-Seq Data before and after trimming. **Supplementary Table 4**. A: Upregulated genes in tumor tissue with log2 Fold Change greater than 1 and Adjusted *p*-value less than 0.01. B: Downregulated genes in tumor tissue with log2 Fold Change less than -1 and Adjusted *p*-value less than 0.01. **Supplementary Table 5.** Comparison of the DEGs from GEO Datasets of GC as well as from DEGs from RNA-Seq Data. 5A: Upregulated Genes. 5B: Down Regulated Genes.

**Additional file 2: Figure S1.** Principal Component Analysis of the samples performed in DESeq. 2.52% and 31% variance were observed on PC1 and PC2, respectively. **Figure S2.** Heatmap showing the expression level of the common DEGs in the tissue samples. The plot is divided in each clustering level.

## Data Availability

The aligned reads in BAM format were submitted European Nucleotide Archive (ENA) at EMBL-EBI under the study accession number PRJEB45410 (URL: https://www.ebi.ac.uk/ena/browser/view/PRJEB45410).

## Abbreviations

STAD: Stomach adenocarcinoma
cDNA: Complementary deoxyribonucleic acid
rRNA: Ribosomal ribonucleic acid
PCR: Polymerase chain reaction
GEO: Gene Expression Omnibus
OS: Overall survival
DEG: Differentially expressed gene
KM Plotter: Kaplan-Meier Plotter
