# Supplementary material for: Transcriptome analysis reveals SALL4 as a prognostic key gene in gastric adenocarcinoma
Source: J Egypt Natl Canc Inst. 2022 Mar 14;34:11. doi: 10.1186/s43046-022-00108-5 (PMC13314243; doi:10.1186/s43046-022-00108-5)
Supplement: Supplementary file 1 — Additional file 1: Supplementary Table 1. Summary of Clinical information of patients included in the study. Supplementary Table 2. Concentration, Purity, and Integrity of Total RNA extracted from Adjacent Normal (D) and Tumor (T) tissues. Supplementary Table 3. Summary of RNA-Seq Data before and after trimming. Supplementary Table 4. A: Upregulated genes in tumor tissue with log2 Fold Change greater than 1 and Adjusted p-value less than 0.01. B: Downregulated genes in tumor tissue with log2 Fold Change less than -1 and Adjusted p-value less than 0.01. Supplementary Table 5. Comparison of the DEGs from GEO Datasets of GC as well as from DEGs from RNA-Seq Data. 5A: Upregulated Genes. 5B: Down Regulated Genes. [file 43046_2022_108_MOESM1_ESM.pdf]

<u>**Supplementary Figures**</u>

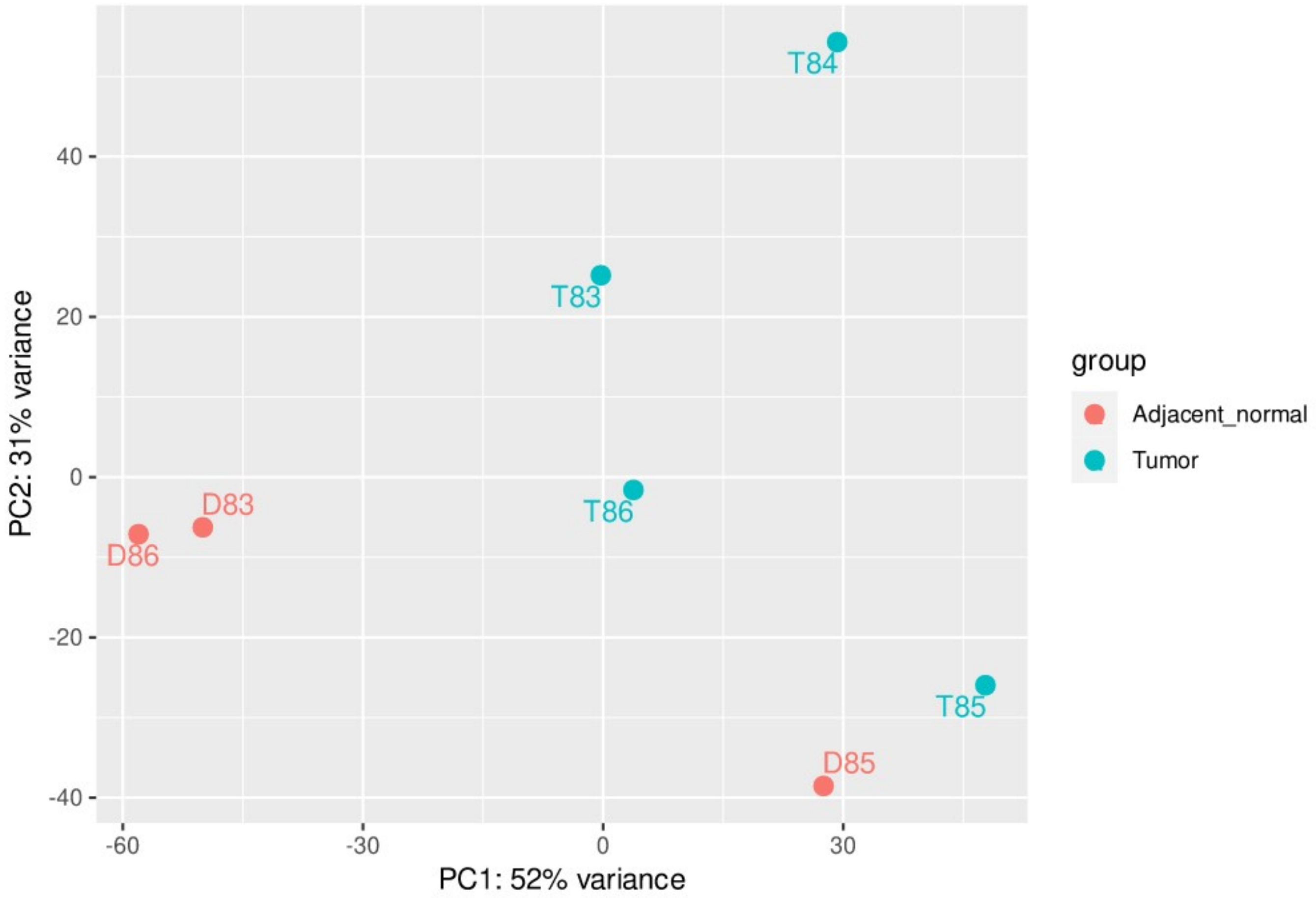

Supplementary Figure 1: Principal Component Analysis of the samples performed in DESeq. 2. 52% and 31% variance were observed in PC1 and PC2, respectively.

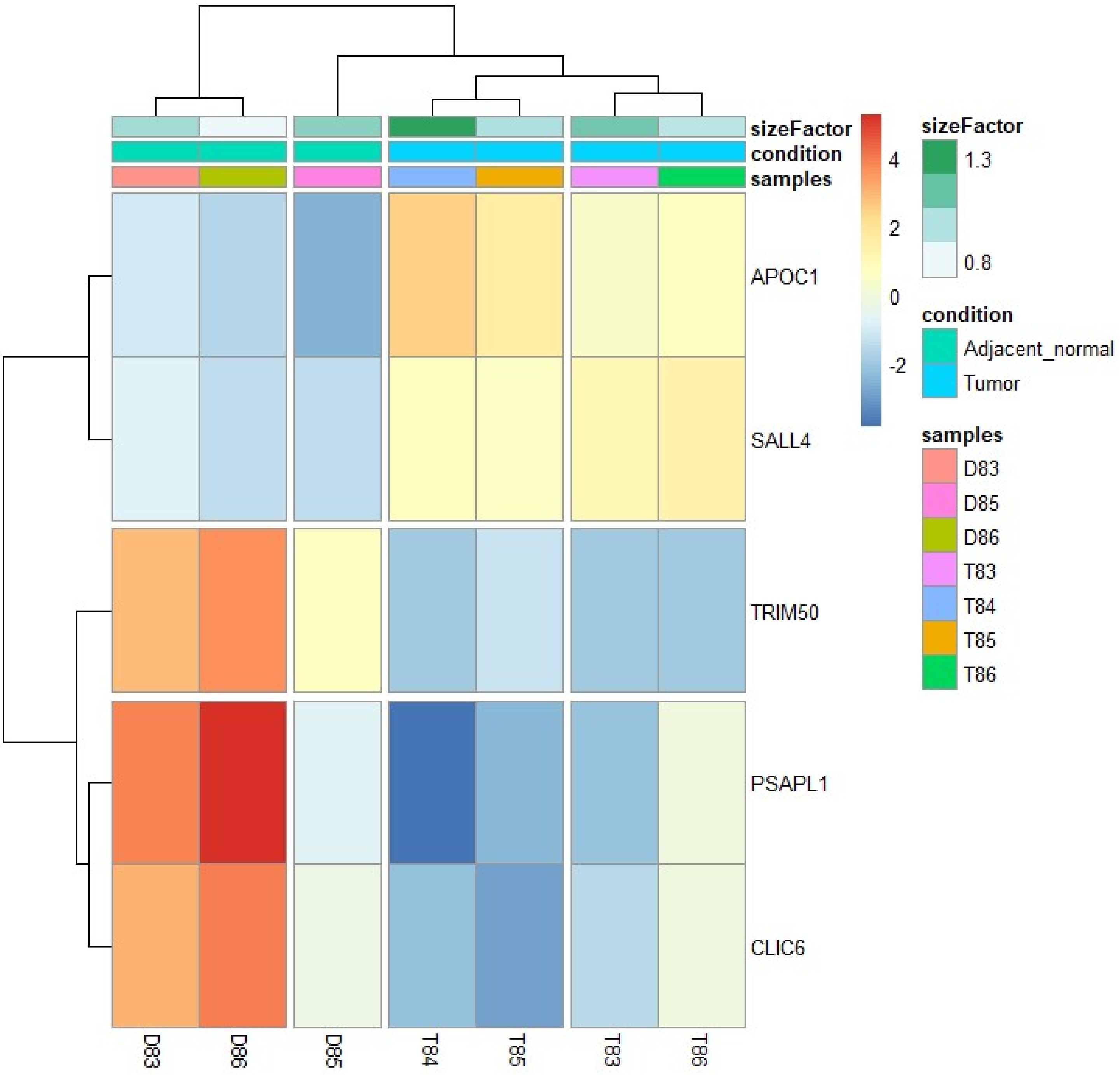

Supplementary Figure 2: Heatmap showing the expression level of the common DEGs in the tissue samples. The plot is divided in each clustering level.
