# Supplementary material for: Transcriptome analysis reveals SALL4 as a prognostic key gene in gastric adenocarcinoma
Source: J Egypt Natl Canc Inst. 2022 Mar 14;34:11. doi: 10.1186/s43046-022-00108-5 (PMC13314243; doi:10.1186/s43046-022-00108-5)
Supplement: Supplementary file 2 — Additional file 2: Figure S1. Principal Component Analysis of the samples performed in DESeq. 2.52% and 31% variance were observed on PC1 and PC2, respectively. Figure S2. Heatmap showing the expression level of the common DEGs in the tissue samples. The plot is divided in each clustering level. [file 43046_2022_108_MOESM2_ESM.pdf]

Supplementary Table 1: Summary of Clinical information of patients included in the study.

Sample ID		Histological grade	Gender	Age (years)	TNM staging
**Adjacent Normal**	**Tumor**				
D83	T83	Moderately differentiated adenocarcinoma	Male	61	T_2 N_2 M_0
D84	T84	Moderately differentiated adenocarcinoma	Female	58	T_{4a} N_{3a} M_0
D85	T85	Poorly differentiated adenocarcinoma	Male	65	T_2 N_0M_0
D86	T86	Poorly differentiated adenocarcinoma	Female	45	T_{4a} $N_{3a}M_0$

D - Distal portion of the Tumor; T- Tumor Tissue.

Supplementary Table 2: Concentration, Purity, and Integrity of Total RNA extracted from Adjacent Normal (D) and Tumor (T) tissues.

Sample ID	rRNA Ratio [28s/18s]	RIN
T83	1.2	7
T84	1.2	8.6
T85	1.3	7.5
T86	1	6.3
D83	1	7.7
*D84	0.6	4.7
D85	1.1	7.1
D86	1.3	6.9

*RNA Sequencing was not performed on the D84 sample ID due to the lower RIN.

Supplementary Table 3: Summary of RNA-Seq Data before and after trimming.

<table>
<tr><th rowspan="2">Sample ID</th><th colspan="3">Before Trimming</th><th colspan="3">After Trimming</th></tr>
<tr><th>Total Reads</th><th>GC Content (%)</th><th>Adapter</th><th>Total Reads</th><th>GC Content</th><th>Adapter</th></tr>
<tr><td>D83_read-1</td><td rowspan="2">19469977</td><td rowspan="2">46</td><td rowspan="14">Yes</td><td rowspan="2">17176911</td><td rowspan="2">45</td><td rowspan="14">No</td></tr>
<tr><td>D83_read-2</td></tr>
<tr><td>D85-read-1</td><td rowspan="2">17385748</td><td rowspan="2">47</td><td rowspan="2">15614115</td><td rowspan="2">47</td></tr>
<tr><td>D85-read-2</td></tr>
<tr><td>D86-read-1</td><td rowspan="2">19289000</td><td rowspan="2">47</td><td rowspan="2">17269912</td><td rowspan="2">46</td></tr>
<tr><td>D86-read-2</td></tr>
<tr><td>T83-read-1</td><td rowspan="2">19561401</td><td rowspan="2">45</td><td rowspan="2">17266844</td><td rowspan="2">46</td></tr>
<tr><td>T83-read-2</td></tr>
<tr><td>T84-read-1</td><td rowspan="2">18595426</td><td rowspan="2">45</td><td rowspan="2">16913754</td><td rowspan="2">45</td></tr>
<tr><td>T84-read-2</td></tr>
<tr><td>T85-read-1</td><td rowspan="2">15656665</td><td rowspan="2">45</td><td rowspan="2">14454534</td><td rowspan="2">44</td></tr>
<tr><td>T85-read-2</td></tr>
<tr><td>T86-read-1</td><td rowspan="2">16685764</td><td rowspan="2">44</td><td rowspan="2">15474623</td><td rowspan="2">43</td></tr>
<tr><td>T86-read-2</td></tr>
</table>

Forward read (read-1) and Reverse read (read-2).
Average number of reads before and after trimming is 18 million and 16 million reads approximately.

Supplementary Table 4A: Upregulated genes in tumor tissue with log2 Fold Change greater than 1 and Adjusted p-value less than 0.01.

Sl. No.	Gene ID	Gene Name	Adjusted P-Value	Log2 Fold Change
1.	ENSG00000169876	*MUC17*	2.88E-05	4.294583
2.	ENSG00000167895	*TMC8*	5.19E-05	1.961363
3.	ENSG00000188404	*SELL*	6.04E-05	1.625197
4.	ENSG00000119547	*ONECUT2*	7.07E-05	4.454432
5.	ENSG00000136231	*IGF2BP3*	9.42E-05	4.055264
6.	ENSG00000007171	*NOS2*	0.000129	6.855044
7.	ENSG00000179869	*ABCA13*	0.00017	5.119563
8.	ENSG00000127152	*BCL11B*	0.000263	1.638227
9.	ENSG00000156510	*HKDC1*	0.000449	4.559387
10.	ENSG00000130208	*APOC1*	0.000482	3.54104
11.	ENSG00000198829	*SUCNR1*	0.000494	3.062318
12.	ENSG00000162892	*IL24*	0.000747	5.380457
13.	ENSG00000181885	*CLDN7*	0.000925	5.217993
14.	ENSG00000254290	*AC124067.4*	0.000935	7.319835
15.	ENSG00000128422	*KRT17*	0.000935	7.593613
16.	ENSG00000125207	*PIWIL1*	0.001098	8.709206
17.	ENSG00000137673	*MMP7*	0.001098	5.439836

18.	ENSG00000143387	*CTSK*	0.001098	2.015552
19.	ENSG00000165215	*CLDN3*	0.00136	5.668342
20.	ENSG00000102854	*MSLN*	0.001577	7.868211
21.	ENSG00000101115	*SALL4*	0.002128	5.280349
22.	ENSG00000106031	*HOXA13*	0.002164	7.009988
23.	ENSG00000012779	*ALOX5*	0.002404	1.277475
24.	ENSG00000111348	*ARHGDIB*	0.002878	1.351204
25.	ENSG00000151651	*ADAM8*	0.003256	1.781929
26.	ENSG00000123388	*HOXC11*	0.003922	6.73439
27.	ENSG00000180745	*CLRN3*	0.003922	5.409425
28.	ENSG00000095970	*TREM2*	0.004554	3.020275
29.	ENSG00000138795	*LEF1*	0.004642	2.292135
30.	ENSG00000107742	*SPOCK2*	0.004642	1.106713
31.	ENSG00000170439	*METTL7B*	0.004642	3.039491
32.	ENSG00000138964	*PARVG*	0.004674	1.548284
33.	ENSG00000140678	*ITGAX*	0.00537	2.351873
34.	ENSG00000179813	*FAM216B*	0.005519	5.363049
35.	ENSG00000101670	*LIPG*	0.005551	1.67937
36.	ENSG00000228742	*LINC02577*	0.005686	6.114184
37.	ENSG00000133048	*CHI3L1*	0.006464	3.78844
38.	ENSG00000228630	*HOTAIR*	0.006464	6.882127

39.	ENSG00000015413	*DPEP1*	0.006494	3.168862
40.	ENSG00000139540	*SLC39A5*	0.007123	6.544985
41.	ENSG00000163347	*CLDN1*	0.00713	3.5484
42.	ENSG00000135362	*PRR5L*	0.007544	1.270048
43.	ENSG00000287151	*C2orf27A*	0.007845	1.806783
44.	ENSG00000118785	*SPP1*	0.007845	5.684947
45.	ENSG00000104894	*CD37*	0.008163	1.943727
46.	ENSG00000106384	*MOGAT3*	0.008226	5.770016
47.	ENSG00000164935	*DCSTAMP*	0.008226	7.397182
48.	ENSG00000136286	*MYO1G*	0.008873	1.56248
49.	ENSG00000142512	*SIGLEC10*	0.008945	2.145621
50.	ENSG00000180818	*HOXC10*	0.009119	7.813677
51.	ENSG00000151790	*TDO2*	0.009553	3.955386
52.	ENSG00000228168	*HNRNPA1P21*	0.00958	5.693809
53.	ENSG00000153976	*HS3ST3A1*	0.009754	2.376582
54.	ENSG00000204252	*HLA-DOA*	0.009754	1.726272
55.	ENSG00000086730	*LAT2*	0.009854	1.814167

Supplementary Table 4B: Downregulated genes in tumor tissue with log2 Fold Change less than -1 and Adjusted p-value less than 0.01.

Sl. No.	Gene ID	Gene Name	Adjusted p-Value	Log2 Fold Change
1.	ENSG00000105675	*ATP4A*	6.58E-18	-13.186
2.	ENSG00000186009	*ATP4B*	7.57E-14	-12.6232
3.	ENSG00000134812	*CBLIF*	1.76E-13	-10.8713
4.	ENSG00000256713	*PGA5*	6.27E-11	-11.7526
5.	ENSG00000158516	*CPA2*	5.33E-10	-10.1322
6.	ENSG00000171560	*FGA*	8.08E-10	-10.3405
7.	ENSG00000205364	*MT1M*	8.08E-10	-3.87745
8.	ENSG00000153303	*FRMD1*	8.08E-10	-8.02121
9.	ENSG00000134216	*CHIA*	4.11E-09	-10.0266
10.	ENSG00000153822	*KCNJ16*	6.70E-09	-8.5773
11.	ENSG00000146755	*TRIM50*	2.22E-07	-8.81315
12.	ENSG00000287725	*AP003071.5*	7.22E-07	-6.22532
13.	ENSG00000284713	*SMIM38*	7.80E-07	-7.20583
14.	ENSG00000170561	*IRX2*	1.78E-06	-6.13899
15.	ENSG00000187957	*DNER*	3.03E-06	-5.81844
16.	ENSG00000105641	*SLC5A5*	3.03E-06	-6.46223
17.	ENSG00000283132	*AC006453.1*	9.06E-06	-8.18684

**18.**	ENSG00000164089	*ETNPPL*	9.38E-06	-8.68647
**19.**	ENSG00000280228	*AC079753.1*	1.12E-05	-5.58278
**20.**	ENSG00000147606	*SLC26A7*	3.60E-05	-5.27089
**21.**	ENSG00000167080	*B4GALNT2*	7.07E-05	-7.31457
**22.**	ENSG00000213373	*LINC00671*	8.90E-05	-7.81118
**23.**	ENSG00000166828	*SCNN1G*	0.000126	-7.1492
**24.**	ENSG00000105290	*APLP1*	0.000129	-4.32387
**25.**	ENSG00000161640	*SIGLEC11*	0.000196	-4.55283
**26.**	ENSG00000132854	*KANK4*	0.000209	-3.22644
**27.**	ENSG00000184368	*MAP7D2*	0.000214	-5.51597
**28.**	ENSG00000235584	*AC008268.1*	0.00022	-8.86443
**29.**	ENSG00000260912	*AL158206.1*	0.000222	-4.26632
**30.**	ENSG00000101210	*EEF1A2*	0.000282	-4.57202
**31.**	ENSG00000280257	*AC007741.1*	0.000282	-8.04312
**32.**	ENSG00000214456	*PLIN5*	0.000339	-3.63642
**33.**	ENSG00000244734	*HBB*	0.000343	-2.7353
**34.**	ENSG00000174514	*MFSD4A*	0.000423	-4.9804
**35.**	ENSG00000187288	*CIDEC*	0.000648	-3.35056
**36.**	ENSG00000177076	*ACER2*	0.000709	-3.93855
**37.**	ENSG00000164850	*GPER1*	0.000747	-2.98411

**38.**	ENSG00000186510	*CLCNKA*	0.000935	-6.12771
**39.**	ENSG00000167653	*PSCA*	0.001028	-6.34598
**40.**	ENSG00000286662	*AC087894.3*	0.001178	-7.0468
**41.**	ENSG00000136826	*KLF4*	0.001272	-2.49131
**42.**	ENSG00000107295	*SH3GL2*	0.001504	-5.01869
**43.**	ENSG00000157315	*TMED6*	0.001706	-3.46083
**44.**	ENSG00000180152	*XIAPP3*	0.002103	-5.25488
**45.**	ENSG00000173597	*SULT1B1*	0.002103	-4.21996
**46.**	ENSG00000122574	*WIPF3*	0.002255	-2.50215
**47.**	ENSG00000159212	*CLIC6*	0.002416	-4.67521
**48.**	ENSG00000101489	*CELF4*	0.002416	-3.34735
**49.**	ENSG00000135549	*PKIB*	0.00244	-3.54719
**50.**	ENSG00000244968	*LIFR-AS1*	0.002624	-2.6261
**51.**	ENSG00000204929	*AC007389.1*	0.00283	-3.68348
**52.**	ENSG00000171885	*AQP4*	0.003086	-9.97827
**53.**	ENSG00000151632	*AKR1C2*	0.00311	-4.49633
**54.**	ENSG00000169605	*GKN1*	0.003401	-6.53516
**55.**	ENSG00000177133	*PRDM16-DT*	0.003548	-3.8705
**56.**	ENSG00000169752	*NRG4*	0.003548	-3.67192
**57.**	ENSG00000274588	*DGKK*	0.003755	-7.29602

**58.**	ENSG00000165192	*ASB11*	0.003922	-7.19316
**59.**	ENSG00000225117	*ARSDP1*	0.00394	-6.42767
**60.**	ENSG00000275395	*FCGBP*	0.004239	-5.16656
**61.**	ENSG00000149809	*TM7SF2*	0.004554	-2.97847
**62.**	ENSG00000168447	*SCNN1B*	0.004642	-4.88781
**63.**	ENSG00000182333	*LIPF*	0.004642	-7.68287
**64.**	ENSG00000206172	*HBA1*	0.004642	-2.90497
**65.**	ENSG00000232044	*SILC1*	0.004876	-5.83349
**66.**	ENSG00000249343	*LINC01333*	0.004876	-7.52565
**67.**	ENSG00000159197	*KCNE2*	0.005051	-5.89077
**68.**	ENSG00000196482	*ESRRG*	0.005302	-6.04947
**69.**	ENSG00000125144	*MT1G*	0.005449	-4.03121
**70.**	ENSG00000174236	*REP15*	0.006036	-4.34673
**71.**	ENSG00000205362	*MT1A*	0.006037	-2.5426
**72.**	ENSG00000171916	*LGALS9C*	0.006794	-4.4575
**73.**	ENSG00000165125	*TRPV6*	0.006821	-3.48945
**74.**	ENSG00000230572	*AC027612.2*	0.007123	-5.55375
**75.**	ENSG00000139988	*RDH12*	0.007473	-4.20108
**76.**	ENSG00000244137	*AL512328.1*	0.007544	-4.20854
**77.**	ENSG00000211445	*GPX3*	0.007544	-1.74358

**78.**	ENSG00000015532	*XYLT2*	0.007722	-2.4528
**79.**	ENSG00000100075	*SLC25A1*	0.007845	-1.10954
**80.**	ENSG00000092068	*SLC7A8*	0.008111	-2.22557
**81.**	ENSG00000130700	*GATA5*	0.008187	-4.37186
**82.**	ENSG00000224511	*LINC00365*	0.008417	-4.64963
**83.**	ENSG00000246763	*RGMB-AS1*	0.009119	-2.7308
**84.**	ENSG00000187689	*AMTN*	0.009439	-4.97514
**85.**	ENSG00000178597	*PSAPL1*	0.009439	-6.08848
**86.**	ENSG00000187527	*ATP13A5*	0.009493	-4.82985
**87.**	ENSG00000162873	*KLHDC8A*	0.009553	-3.53057
**88.**	ENSG00000107159	*CA9*	0.009555	-4.86371
**89.**	ENSG00000286335	*AC009230.1*	0.009625	-4.18926
**90.**	ENSG00000166123	*GPT2*	0.009754	-2.64232
**91.**	ENSG00000118596	*SLC16A7*	0.009754	-2.49732
**92.**	ENSG00000230185	*C9orf147*	0.009824	-3.14298
**93.**	ENSG00000186297	*GABRA5*	0.009854	-4.37209

Supplementary Table 5: Comparison of the DEGs from GEO Datasets of GC as well as from DEGs from RNA-Seq Data.

5A: Upregulated Genes

Names	total	elements
GSE19826<br>GSE79973<br>MZU-GC-Patient	2	*APOC1, SALL4*
GSE19826<br>MZU-GC-Patient	6	*HKDC1, ONECUT2, HOXA13, CLDN1, CLDN7, CTSK*
GSE79973<br>MZU-GC-Patient	2	*IGF2BP3, SPP1*
GSE19826<br>GSE79973	43	*FSTL1, CDH3, SFRP4, SPARC, COL5A1, FNDC1, COL3A1, COL4A1, VCAN, ACTN1, LUM, COL18A1, CTHRC1, TMEM158, CDH11, SULF1, MEST, SERPINH1, COL5A2, FBN1, FAP, BMP1, TIMP1, IGFBP7, THBS2, RAB31, THY1, BGN, INHBA, COL1A2, COL6A3, CST1, MFAP2, COL1A1, ADAMTS2, COL8A1, COL12A1, FN1, TEAD4, LGALS1, COL10A1, PRRX1, ASPN*

5B: Down Regulated Genes:

Names	total	elements
GSE19826<br>GSE79973<br>MZU-GC Patient	3	*PSAPL1, CLIC6, TRIM50*
GSE19826<br>MZU-GC Patient	6	*ASB11, MFSD4A, FRMD1, SH3GL2, WIPF3, PLIN5*
GSE79973<br>MZU-GC Patient	21	*MT1G, CPA2, ETNPPL, CIDEC, SIGLEC11, ATP4A, SCNN1B, SCNN1G, GKN1, RDH12, KCNJ16, SLC26A7, SULT1B1, ATP4B, AQP4, SLC7A8, MT1M, AKR1C2, PKIB, ESRRG, PGA5*
GSE19826<br>GSE79973	15	*SMIM5, SVIP, MYRF, UBL3, AZGP1P1, STX12, AZGP1, ELOVL6, MAGI3, CWH43, ZNF57, RASSF6, TMPRSS2, SMIM6, NFE2L2*

GSE19826 – Microarray dataset of 12 Tumor and 15 adjacent normal tissues analyzed in geo2r.

GSE79973- Microarray dataset of 10 Tumor and 10 adjacent normal tissues analyzed in geo2r.

MZU-GC patient - DEGs from RNA-Seq data .
